# A Collaborative Scheduling Model for the Supply-Hub with Multiple Suppliers and Multiple Manufacturers

**DOI:** 10.1155/2014/894573

**Published:** 2014-04-15

**Authors:** Guo Li, Fei Lv, Xu Guan

**Affiliations:** ^1^School of Management and Economics, Beijing Institute of Technology, Beijing 100081, China; ^2^School of Management, Huazhong University of Science and Technology, Wuhan 430074, China; ^3^Economics and Management School, Wuhan University, Wuhan 430072, China

## Abstract

This paper investigates a collaborative scheduling model in the assembly system, wherein multiple suppliers have to deliver their components to the multiple manufacturers under the operation of Supply-Hub. We first develop two different scenarios to examine the impact of Supply-Hub. One is that suppliers and manufacturers make their decisions separately, and the other is that the Supply-Hub makes joint decisions with collaborative scheduling. The results show that our scheduling model with the Supply-Hub is a NP-complete problem, therefore, we propose an auto-adapted differential evolution algorithm to solve this problem. Moreover, we illustrate that the performance of collaborative scheduling by the Supply-Hub is superior to separate decision made by each manufacturer and supplier. Furthermore, we also show that the algorithm proposed has good convergence and reliability, which can be applicable to more complicated supply chain environment.

## 1. Introduction

To avoid the supply delay risk caused by any supplier, in practice, manufacturer/assembler normally prefers to outsource its purchasing business to the third party logistics (TPL) and requires his suppliers to hold inventory in the warehouse operated by TPL. Given this trend, a new type of TPL arises in recent years, which is called Supply-Hub. Investigated by many scholars [1–3], the Supply-Hub is an integrated logistics provider with a series of logistic services (e.g., assemblage, distribution, and warehouse), which is widely applied in the auto and electronic industries to support the manufacturer to implement the JIT (Just In Time) production, so as to respond to the market changes rapidly. For example, BAX GLOBAL and UPS are two typical logistic companies operated under Supply-Hub mode with Chinese auto companies.

For most Supply-Hubs, they are located near the manufacturer's factory, so as to store most of the raw materials delivered by the suppliers. According to the agreement, the Supply-Hub will charge the suppliers for the components consumed during a fixed period of time. However, during this process, it is very hard for the Supply-Hub to coordinate the production and the delivery among different suppliers, specific to how to precisely determine the supplier's production lot, the distribution frequency, and the distribution quantity. In actual business activities, when the material flows are sufficiently large, the coordination and optimization of production and distribution based on the Supply-Hub can bring substantial benefits to the members in the supply chain.

Our paper is related to the vast literature, which can be divided into two groups. The first group concerns the order allocation, vehicle routing, and production planning. Hahm and Yano [4] explore the economic lot and delivery scheduling problem. Moreover, Hahm and Yano [5, 6], Khouja [7], and Clausen and Ju [8] consider the problems of determining the production scheduling and distribution intervals for different types of components when a supplier provides different kinds of components. Vergara et al. [9] propose the genetic algorithm to make production scheduling and cycle time arrangements for many kinds of components in a simple multistage supply chain, where each supplier provides one or a variety of products for the upstream supplier or assembler. Khouja [10] examines production sequencing and distribution scheduling in a single-product and multi-product supply chain when production intervals equal distribution intervals. However, all of the above literature focus on a simple supply chain structure, and the problems of production and distribution in an assembly supply chain with multiple suppliers and multiple manufacturers have not been involved. Pundoor [11] first establishes a cooperative scheduling model of production and distribution in a multi-suppliers, one-warehouse, and one-customer system. The supplier's production and distribution interval and warehouse's distribution interval are collaboratively optimized to minimize the unit production and logistics cost in the upstream supply chain wherein supplier's production capability is limited. However, the model does not consider the transportation constraint from the warehouse to the manufacturer. Torabi et al. [12] investigate the lot and delivery scheduling problem in a simple supply chain where a single supplier produces multiple components on a flexible flow line (FFL) and delivers them directly to an assembly facility (AF). They also develop a new mixed integer nonlinear program (MINLP) and an optimal enumeration method to solve the problem. Naso et al. [13] focus on the ready-mixed concrete delivery. They propose a novel meta-heuristic approach based on a hybrid genetic algorithm combined with constructive heuristics. Ma and Gong [14] extend the work of Pundoor [11] to a multi-suppliers and one-manufacturer system based on the Supply-Hub. In their model the production lot and distribution interval are optimized from either supplier's or manufacturer's perspective.

The second group is about the coordination with the application of Supply-Hub. Barnes et al. [1] find that the Supply-Hub is an innovative strategy to reduce cost and improve responsiveness. They further define the concept of Supply-Hub and review its development by analyzing the practical case. They also propose a prerequisite of establishing Supply-Hub and the main way of operating Supply-Hub. Shah and Goh [3] explore the operation strategy of Supply-Hub to achieve the joint operation management between customers and upstream suppliers. Moreover, they analyze how to manage the supply chain better in a vendor-managed inventory model. Furthermore, they find that the relationships between the operation strategies and performance evaluations of Supply-Hub are complex and nonlinear. As a result, they propose a hierarchical structure to help the Supply-Hub achieve the balance among different members. Lin and Chen [15] propose a generalized hub-and-spoke network in a capacitated and directed network configuration that integrates the operations of three common hub-and-spoke networks: pure, stopover, and center directs. They also develop an implicit enumeration algorithm with embedded integrally constrained multicommodity min-cost flow. Lin [16] studies the integrated hierarchical hub-and-spoke network design problem for dual services. They propose a directed network configuration and formulate a link-based integer mathematical model, and also develop a link-based implicit enumeration with an embedded degree and time constrained spanning tree algorithm. Charles et al. [17] investigate how implement integrated logistics hubs by considering six independent industrial sectors with reference models and systems. The research results provide a field tested method for deriving integrated logistics hub models in different manufacturing economies with notes that provide sufficient methodological details for repeating the construction of logistics hubs in other manufacturing economies.

Based on the Supply-Hub, Ma and Gong [14] develop collaborative decision-making models of production and distribution considering the matching of distribution quantity between suppliers. The result shows that the total supply chain cost and the production cost of suppliers decrease significantly, but the logistics cost of manufacturers and the operational cost of Supply-Hub increase. In order to explore the effect of the supply chain design caused by the structural changes in the assembly system, Li et al. [18] establish several supply chain design models (one without supply center, one with single-stage supply center, and one with two-stage supply center) according to the characteristics of bill of material (BOM) and the relationships of multiple properties among suppliers. With the consideration that multiple suppliers provide different components to a manufacturer based on the Supply-Hub, Gui and Ma [19] establish an economical order quantity model in such two ways as picking up separately from different suppliers and milk-run picking up. The result shows that the sensitivity to carriage quantity of the transportation cost and the demand variance in different components have an influence on the choices of two picking up ways. Li et al. [20] give a thorough review about collaborative operation and optimization in supply logistics based on the Supply-Hub and point out that how to coordinate suppliers and share risks is still to be explored.

Obviously, the above literature does not take the coordination issue of Supply-Hub into account. In the actual operation process of Supply-Hub, the service for multiple suppliers and multiple manufacturers are often provided by a single Supply-Hub. For example, BAX GLOBAL takes in charge of the logistics services in Southeast Asia for Apple, Dell, and IBM. From the perspective of Supply-Hub, how to integrate resources of multiple suppliers and multiple manufacturers is the key point of implementing JIT policy in the supply chain.

## 2. Problem Definition and Notation

### 2.1. Problem Description

**Figure 1:**
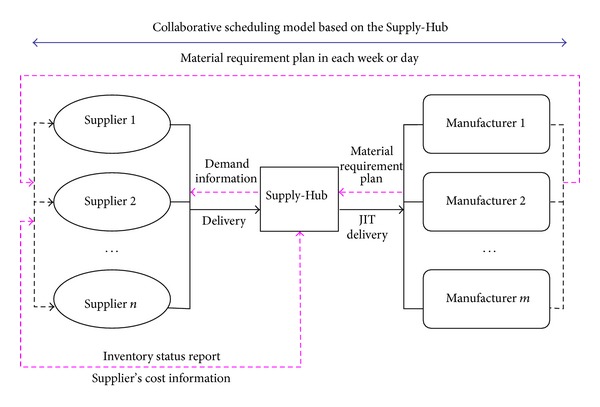
Multi-suppliers and multi-manufacturers operation mode based on the Supply-Hub.

Let us consider the following operation process: each manufacturer sends its material requirement plan to the Supply-Hub and corresponding suppliers based on a rolling plan. After that, the Supply-Hub optimizes and arranges the production and distribution activities for each supplier based on the information of production costs and inventory status. Finally, the Supply-Hub implements JIT direct-station distribution according to material requirement plan in each week or day provided by each manufacturer. The illustration of the process is shown in Figure 1. It is worth mentioning that the production information is freely shared among the suppliers, the Supply-Hub and the manufacturers.

Note that the coordination scheduling is to implement the JIT distribution of components required by each manufacturer with minimal cost. To achieve this goal, the manufacturer's distribution lot, the supplier's production lot, and the distribution frequency should be optimized through integration of the entire supply chain and logistics operation based on the Supply-Hub. In Figure 1, the Supply-Hub provides the service for *m* manufacturers and *n* suppliers.

For manufacturer *j*, where *j* = 1,2,…, *m*, the number of its suppliers is *k*_*j*_, where 1 ≤ *k*_*j*_ ≤ *n*. It indicates that the components required by manufacturer *j* are provided by *k*_*j*_ suppliers. For a certain supplier *i*, where *i* = 1,2,…, *n*, the number of components required by manufacturer is *l*_*i*_, where 1 ≤ *l*_*i*_ ≤ *m*. It implies that the components provided by supplier *i* are required by *l*_*i*_ manufacturer. Therefore, the multi-suppliers and multi-manufacturers system based on the Supply-Hub considered in our paper is more universal and versatile.

### 2.2. Assumptions and Notations

The Supply-Hub takes charge in the components purchasing and JIT direct-station distribution for *m* manufacturers. Component *i* required by manufacturer *j* is delivered to manufacturer *j* by the Supply-Hub at suitable interval *R*_*h*,*j*_, and component *i* from supplier *i* was delivered to the Supply-Hub at regular interval *R*_*ij*_. According to the distribution lot to the Supply-Hub, the purchasing lot is determined by supplier *i*.

Define

(1)  wij={1if supplier i provides component i for manufacturer j 0else.

#### 2.2.1. Assumptions

The specific assumptions are as follows.

1. Each supplier provides one kind of the component for a manufacturer, and demand for the component is constant. Note that our results remain unchanged if a certain supplier can provide a variety of components, since it can be actually converted to multiple suppliers and each provides one component.
2. The transportation cost of component *i* required by manufacturer *j* from supplier *i* to the Supply-Hub is composed of a fixed cost *F*_*ij*_ and a variable cost *V*_*ij*_, and the transportation cost from the Supply-Hub to manufacturer *j* also contains a fixed cost *F*_*h*,*ij*_ and a variable cost *V*_*h*,*ij*_.
3. The lead time for each level of the supply chain is constant, and it is assumed to be zero without loss of generality.
4. Shortages are not allowed.
5. Time horizon is infinite.

#### 2.2.2. Notations

The input parameters and decision variables for manufacturers, the Supply-Hub, and suppliers, are denoted by the subscripts *m*, *h*, and *s*, respectively.

*Manufacturers:*

 
*m* is the number of manufacturer; where *j* = 1,2,…, *m*
 
*d*_*ij*_ is the annual demand of manufacturer *j* for the component *i* (units/year);
 
*h*_*m*,*ij*_ is the manufacturer *j*'s holding cost per unit per year for component *i*;
 
*A*_*m*,*j*_ is the order cost for manufacturer *j* ($);
 
*T*_*j*_ is the cycle time (year).

*The Supply-Hub:*

 
*A*_*h*_ is the fixed-order/setup cost per cycle for the Supply-Hub;
 
*h*_*h*,*i*_ is the Supply-Hub's holding cost per unit per year for component *i*;
 
*M*_*h*,*j*_ is an integer multiplier to adjust the order quantity of the Supply-Hub to that of manufacturer *j*;
 
*F*_*h*,*j*_ is the fixed transportation cost from the Supply-Hub to manufacturer *j*;
 
*V*_*h*,*j*_ is the variable transportation cost from the Supply-Hub to manufacturer *j*.

*Suppliers:*

 
*n* is the number of suppliers, where *i* = 1,2,…, *n*;
 
*A*_*s*,*i*_ is the order cost for supplier *i*;
 
*h*_*s*,*i*_ is the supplier's holding cost per unit per unit per year for component *i*;
 
*M*_*s*,*ij*_ is an integer multiplier to adjust the order quantity of the supplier *i* whose component is required by manufacturer *j* to that of the Supply-Hub;
 
*F*_*s*,*ij*_ is the fixed transportation cost for component *i* required by manufacturer *j* from supplier *i* to the Supply-Hub;
 
*V*_*s*,*ij*_ is the variable transportation cost for component *i* required by manufacturer *j* from supplier *i* to the Supply-Hub.

## 3. Model Formulation

### 3.1. Manufacturer's Cost Function

Manufacturer *j* orders ∑_*i*=1_^*n*^ *d*_*ij*_ *T*_*j*_ units from the Supply-Hub every *T*_*j*_. The total annual cost for a manufacturer is the sum of the annual order cost, *A*_*m*,*j*_/*T*_*j*_, and the annual holding cost, ∑_*i*=1_^*n*^ *d*_*ij*_ *T*_*j*_ *h*_*m*,*ij*_/2. The annual cost function for manufacturer *j* is given by

(2) $$\begin{array}{c} C_{m,j}=\frac{A_{m,j}}{T_{j}}+\frac{\sum_{i=1}^{n}d_{ij}T_{j}h_{m,ij}}{2}. \end{array}$$

The annual manufacturers' cost is the sum of *C*_*m*,*j*_ for *m* manufacturers, and it is given as

(3) $$\begin{array}{c} C_{m}=\overset{m}{\underset{j=1}{\sum }}C_{m,j}\left(T_{j}\right)=\overset{m}{\underset{j=1}{\sum }}\left(\frac{A_{m,j}}{T_{j}}+\frac{\sum_{i=1}^{n}d_{ij}T_{j}h_{m,ij}}{2}\right), \end{array}$$

where *T*_*j*_ is a decision variable in (3), and the optimal cycle time for manufacturer *j* is

(4) $$\begin{array}{c} T_{j}^{*}=\sqrt{\frac{2A_{m,j}}{\sum_{i=1}^{n}d_{ij}h_{m,ij}}}. \end{array}$$

In this paper, an optimal value of decision variable will be indicated by an asterisk (∗).

### 3.2. Supply-Hub's Cost Function

The Supply-Hub manages its upstream manufacturers separately; thus, it places an order for manufacturer *j* every *M*_*h*,*j*_ *T*_*j*_ and transports the components to manufacturer *j* every *T*_*j*_. The Supply-Hub's annual cost to satisfy the demand of manufacturer *j* is

(5) Ch,j(Mh,j)=AhMh,jTj+∑i=1n[hh,i2(Mh,j−1)dijTj] +Fh,j+∑i=1ndijTjVh,jTj,

where the terms *A*_*h*_/*M*_*h*,*j*_ *T*_*j*_, ∑_*i*=1_^*n*^[(*h*_*h*,*i*_/2)(*M*_*h*,*j*_ − 1)*d*_*ij*_ *T*_*j*_], and (*F*_*h*,*j*_ + ∑_*i*=1_^*n*^ *d*_*ij*_ *T*_*j*_ *V*_*h*,*j*_)/*T*_*j*_ are the Supply-Hub's annual order cost, the holding cost, and transportation cost for manufacturer *m* which requires component *i* from *n* suppliers. Then the Supply-Hub's total cost is the sum of (5) for *m* manufacturers, and it is given as

(6) Ch=∑j=1mCh,j(Mh,j)=∑j=1m{AhMh,jTj+∑i=1n[hh,i2(Mh,j−1)dijTj]   +Fh,j+∑i=1ndijTjVh,jTj},

where *M*_*h*,*j*_ is a decision variable in (6), and the Supply-Hub's optimal cycle time for manufacturer *j* is

(7) $$\begin{array}{c} M_{h,j}^{}=\frac{1}{T_{j}}\sqrt{\frac{2A_{h}}{\sum_{i=1}^{n}h_{h,i}d_{ij}}}. \end{array}$$

### 3.3. Supplier's Cost Function

The Supply-Hub has *n* suppliers to provide all *n* components. When manufacturer *j* places an order of size ∑_*i*=1_^*n*^ *d*_*ij*_ *T*_*j*_ with the Supply-Hub every *T*_*j*_ and as discussed above, the Supply-Hub determines its order quantity *M*_*h*,*j*_ *d*_*ij*_ *T*_*j*_ for the supplier *i*. In order to fulfill the demand of manufacturer *j*, the order of size *M*_*h*,*j*_ *d*_*ij*_ *T*_*j*_ will be placed by the Supply-Hub, and shipment will occur every *M*_*h*,*j*_ *T*_*j*_. The annual cost for supplier *i* is written as

(8) Cs,i(Ms,ij)=∑j=1m[As,iMs,ijMh,jTj+hs,iMh,j2(Ms,ij−1)dijTj   +Fs,ij+Vs,ijMh,jTjdijMh,jTj],

where the terms *A*_*s*,*i*_/*M*_*s*,*ij*_ *M*_*h*,*j*_ *T*_*j*_, (*h*_*s*,*i*_ *M*_*h*,*j*_/2)(*M*_*s*,*ij*_ − 1)*d*_*ij*_ *T*_*ij*_, and (*F*_*s*,*ij*_ + *V*_*s*,*ij*_ *M*_*h*,*j*_ *T*_*j*_ *d*_*ij*_)/*M*_*h*,*j*_ *T*_*j*_ are, respectively, the annual order cost, holding cost, and transportation cost for supplier *i* to meet the annual demand for components required by the Supply-Hub. Then the collective annual cost for *n* suppliers is given as

(9) Cs=∑i=1nCs,i(Ms,ij)=∑i=1n∑j=1m[As,iMs,ijMh,jTj+hs,iMh,j2(Ms,ij−1)dijTj+Fs,ij+Vs,ijMh,jTjdijMh,jTj],

where *M*_*s*,*ij*_ is a decision variable in (9), and the supplier *i*'s optimal cycle time for manufacturer *j* is

(10) $$\begin{array}{c} M_{s,ij}^{}=\frac{1}{M_{h,j}T_{j}}\sqrt{\frac{2A_{s,i}}{d_{ij}}}. \end{array}$$

### 3.4. Solution Procedures with Decentralized Decision

1. Each manufacturer *j* determines its optimal cycle time, $T_{j}^{*}=\sqrt{2A_{m,j}/\sum_{i=1}^{n}d_{ij}h_{m,ij}}$, where *i* = 1,2,…, *n*, *j* = 1,2,…, *m*. Then the collective annual manufacturers' cost *C*_*m*_ is computed from (3).
2. The value of *T*_j_* is input into (7), $M_{h,j}^{}=(1/T_{j}^{*})\sqrt{2A_{h}/\sum_{i=1}^{n}h_{h,i}d_{ij}}$. If *C*_*h*,*j*_(⌈*M*_*h*,*j*_⌉) ≥ *C*_*h*,*j*_(⌊*M*_*h*,*j*_⌋), then *M*_*h*,j_* = ⌊*M*_*h*,*j*_⌋. Or else *M*_*h*,j_* = ⌈*M*_*h*,*j*_⌉. This should be repeated for *m* manufacturers, after which the collective Supply-Hub's annual cost, *C*_*h*_ = ∑_*j*=1_^*m*^ *C*_*h*,*j*_, is computed from (6).
3. The values of *T*_j_* and *M*_*h*,j_* are input into (8), and (8) is minimized by searching the optimal value of *M*_*s*,*ij*_. If *C*_*s*,*i*_(⌈*M*_*s*,*ij*_⌉) ≥ *C*_*s*,*i*_(⌊*M*_*s*,*ij*_⌋), then *M*_*s*,ij_* = ⌊*M*_*s*,*ij*_⌋, or else *M*_*s*,ij_* = ⌈*M*_*s*,*ij*_⌉. This may be repeated for *m* · *n* times because the component *i* provided by supplier *i* may be required by manufacturer *j*, where *i* = 1,2,…, *n*, *j* = 1,2,…, *m*. Then the collective supplier's annual cost *C*_*s*_ is computed from (9).
4. The value of optimal *T*_j_*, *M*_*h*,j_*, and *M*_*s*,ij_* for each side should be recorded and the total supply chain cost for the case of no coordination is *C*_*nsc*_ = *C*_*m*_ + *C*_*h*_ + *C*_*s*_, which can be obtained after the above three steps.

## 4. Supply Chain Coordination

The annual supply chain's cost is determined by summing (3), (6), and (9) to obtain

(11) Ccsc=Cm+Ch+Cs=∑j=1m(Am,jTj+∑i=1ndijTjhm,ij2) +∑j=1m{AhMh,jTj+∑i=1n[hh,i2(Mh,j−1)dijTj]    +Fh,j+∑i=1ndijTjVh,jTj} +∑i=1n∑j=1m[As,iMs,ijMh,jTj+hs,iMh,j2(Ms,ij−1)dijTj     +Fs,ij+Vs,ijMh,jTjdijMh,jTj].

This is a centralized decision-making process, in which the Supply-Hub tries to schedule and optimize each decision variable for the entire supply chain. It is general and practical that the Supply-Hub takes charge of distribution frequency and purchasing frequency for the suppliers and the manufacturers, respectively.

Note that (11) is convex and differentiable over *T*_*j*_, where ∂^2^ *C*_csc_/∂^2^ *T*_*j*_ = ∑_*j*=1_^*m*^[(2/*T*_*j*_^3^)(*A*_*m*,*j*_ + (*A*_*h*_/*M*_*h*,*j*_) + *F*_*h*,*j*_ + ∑_*i*=1_^*n*^(*A*_*s*,*i*_/*M*_*s*,*ij*_ *M*_*h*,*j*_)]+∑_*i*=1_^*n*^(*F*_*s*,*ij*_/*M*_*h*,*j*_)) > 0 for every *T*_*j*_ > 0, since *A*_*m*,*j*_, *A*_*h*_, *F*_*h*,*j*_, *F*_*s*,*ij*_, *A*_*s*,*i*_, *M*_*h*,*j*_, *M*_*s*,*ij*_ > 0. Therefore at a particular set of values for *M*_*s*,*ij*_ ≥ 1 and *M*_*h*,*j*_ ≥ 1, where *M*_*s*,*ij*_ and *M*_*h*,*j*_ are integer, *i* = 1,2,…, *n*, *j* = 1,2,…, *m*, the first derivative of (11) should be set to zero and the optimal *T*_j_* was obtained. Consider

(12) Tj∗=(2[Am,j+AhMh,j+Fh,j+∑i=1nAs,i(Ms,ijMh,j)+∑i=1nFs,ijMh,j]×(∑i=1ndijhm,ij+(Mh,j−1)∑i=1nhh,idij+Mh,j×∑i=1n(Ms,ij−1)dij)−1)1/2.

### 4.1. Complexity Analysis for This Problem

The complexities of solving this problem are analyzed as follows. The optimal *T*_*j*_, *M*_*h*,*j*_, and *M*_*s*,*ij*_ should be obtained to minimize the supply chain's cost *C*_csc_, where *i* = 1,2,…, *n*, *j* = 1,2,…, *m*. If a certain group of solution to this problem was proved NP-complete, then the whole group of solutions to this problem must be NP-complete.

Taking supplier 1 as the representative case, whose problem is to minimize *C*_csc_(*M*_*s*,1*j*_, *M*_*h*,*j*_, *T*_*j*_ | *j* = 1,2,…, *m*). We define this problem as *P*. If the problem *P* can be proved to equal partition problem, then the problem *P* is NP-complete.

*Partition Problem.* Given the positive integer *n*, *B*, and a group of positive integers *G* = {*x*_1_, *x*_2_,…, *x*_*n*_}, then ∑_*i*=1_^*n*^ *x*_*i*_ = 2*B*, can *G* be divided into group *G*_1_ and *G* − *G*_1_ to make ∑_*x*_*i*_∈*G*_*i*__ *x*_*i*_ = ∑_*x*_*i*_∈*G*−*G*_*i*__ *x*_*i*_ = *B*.

**Lemma 1**

Partition is NP-complete; see Garey and Johnson [21].

**Proposition 2**

The problem *P* is NP-complete.

**Proof**

We should transform the problem *P* to partition. Let the sets *T*, *M*_*h*_, *M*_*s*,1_, with |*T*| = |*M*_*h*_| = |*M*_*s*,1_| = *m*, and *W*⊆*T* × *M*_*h*_ × *M*_*s*,1_ be an arbitrary instance of problem *P*. Let the elements of these sets be denoted by *T* = {*T*_1_, *T*_2_,…, *T*_*m*_}, *M*_*h*_ = {*M*_*h*,1_, *M*_*h*,2_,…, *M*_*h*,*m*_}, *M*_*s*,1_ = {*M*_*s*,11_, *M*_*s*,12_,…, *M*_*s*,1*m*_}, and *W* = {*W*_1_, *W*_2_,…, *W*_*q*_}, where |*W*| = *q*. We should construct a set *G* and a size *s* (*a*) ∈ *Z* ^+^ for each *a* ∈ *G*, such that *G* contains a subset *G*_1_ satisfying

(13) $$\begin{array}{c} \overset{}{\underset{a\in G_{1}}{\sum }}s\left(a\right)=\overset{}{\underset{a\in G-G_{1}}{\sum }}s\left(a\right). \end{array}$$

The set *G* will contain a total of *q* + 2 elements and will be constructed in two steps.

The first *q* elements of *G* are {*a*_*k*_ : 1 ≤ *k* ≤ *q*}, where the element *a*_*k*_ is associated with the group *W*_*k*_ ∈ *W*. The size *s* (*a*_*k*_) of *a*_*k*_ will be specified by giving its binary representation, in terms of a string of 0's and 1's divided into 3*m* “zones” of *p* = [log_2_(*q* + 1)] bits each.

Then each *s* (*a*_*k*_) can be expressed in binary with no more than 3*pm* bits; it is clear that *s* (*a*_*k*_) can be constructed from the given problem *P* instance in polynomial time; see Garey and Johnson [21].

If we sum up all elements in any zone, the total can never exceed *q* = 2^*p*^ − 1. Therefore, in adding up ∑_*a*∈*G*_1__ *s* (*a*) for any subset *G*_1_ ∈ {*a*_*k*_ : 1 ≤ *k* ≤ *q*}, there will never be any “carries” from one zone to the next. If we define *B* = ∑_*s*=0_^3*m*−1^2^*ps*^, then any subset *G*_1_ ∈ {*a*_*k*_ : 1 ≤ *k* ≤ *q*} will satisfy

(14) $$\begin{array}{c} \overset{}{\underset{a\in G_{1}}{\sum }}s\left(a\right)=B. \end{array}$$

The last two elements are denoted by *b*_1_ and *b*_2_; that is,

(15) $$\begin{array}{c} s\left(b_{1}\right)=2\overset{q}{\underset{k=1}{\sum }}s\left(a_{k}\right)-B,\\ s\left(b_{2}\right)=\overset{q}{\underset{k=1}{\sum }}s\left(a_{k}\right)+B. \end{array}$$

Now suppose we have a subset *G*_1_ ∈ *G* such that

(16) $$\begin{array}{c} \overset{}{\underset{a\in G_{1}}{\sum }}s\left(a\right)=\overset{}{\underset{a\in G-G_{1}}{\sum }}s\left(a\right). \end{array}$$

Then both of these sums must be equal to 2∑_*k*=1_^*q*^ *s* (*a*_*k*_), and one of the two sets, *G*_1_ or *G* − *G*_1_, contains *b*_1_ but not *b*_2_. It follows that the remaining elements of that set form a subset of {*a*_*k*_ : 1 ≤ *k* ≤ *q*} whose sizes sum to *B*. Therefore the problem *P* can be transformed to partition, and Proposition 2 is proved.

### 4.2. Solution Procedure

Since the coordination scheduling problem of multiple suppliers and multiple manufacturers based on the Supply-Hub is NP-complete, the solution may be very complex. Therefore, the auto-adapted differential evaluation algorithm will be proposed to solve this problem by this paper. The differential evolution algorithm put forward by Rainer Storn and Kenneth Price in 1997 is for meta-heuristic global optimization based on population evolutionary and the real coding, which is originally used to solve the Chebyshev polynomials. As to more complex global optimization problems of continuous space, such as non-linear and nondifferentiable problems even without function expression, the differential evaluation algorithm has a better global optimization ability and higher convergence performance with simple operation, less controlling parameters, and better robustness, compared to genetic algorithms, particle swarm optimization, simulated annealing, tabu search, and so forth.

The evolution process of differential evaluation algorithm is similar to genetic algorithms, including population initialization, variation, hybridization, and selection. But the main differences between these two algorithms are that the process of variation is before hybridization for differential evolution algorithm, and evaluation of population depends on comparisons with testing chromosome and target chromosome. As a result, the solution procedure of coordination scheduling problem can be proposed as follows.

#### 4.2.1. Population Initialization

Let *g* stands for the generation of population *P* ^*g*^, and the scale of population is NP; that is, *P* ^*g*^ = {*x*_i*_^g^}, where i* = 1,2,…, NP. *x*_i*_^g^ is a feasible solution of the population *P* ^*g*^, which is composed of a vector of *D* variables, that is *x*_i*_^g^ = (*x*_i*1_^*g*^, *x*_i*2_^*g*^,…, *x*_i*D_^*g*^).

As for our scheduling problem, *D* is the number of decision variables. Let *x*_i*_^g^ = (*x*_i*1_^*g*^, *x*_i*2_^*g*^,…, *x*_i*D_^*g*^) = (*M*_*h*,1_, *M*_*h*,2_,…, *M*_*h*,*m*_; *M*_*s*,11_, *M*_*s*,21_,…, *M*_*s*,*n*1_; *M*_*s*,12_, *M*_*s*,22_,…, *M*_*s*,*n*2_; …; *M*_*s*,1*m*_,*M*_*s*,2*m*_,…, *M*_*s*,*nm*_).

Initialize the population, set *g* = 0, and *x*_i*j*_^g=0^ = *l*_j*_ + rand_j*_ · (*h*_j*_ − *l*_j*_).

Where rand_j*_ is a real number generated by uniform random distribution in [0,1), *h*_j*_ and *l*_j*_ are the upper and lower boundaries of individual variables, which are randomly distrusted real numbers.

#### 4.2.2. Variations

The interim of individuals, *v*_i*_^g+1^ = (*v*_i*1_^*g*+1^, *v*_i*2_^*g*+1^,…, *v*_i*D_^*g*+1^), should be generated after any individual *x*_i*_^g^ is determined in population *P* ^*g*^, where the number of *x*_i*_^g^ is *r* (3 ≤ *r* ≤ NP). Let individual set *Ω* = {*ξ*_1_, *ξ*_2_,…, *ξ*_*r*_} and after variation the interim individuals *v*_i*_^g+1^ are

(17) $$\begin{array}{c} v_{i^{*}}^{g+1}=\xi_{1}+F\cdot \left[\left(\xi_{2}-\xi_{3}\right)+\left(\xi_{3}-\xi_{4}\right)+\cdots +\left(\xi_{r-1}-\xi_{r}\right)\right], \end{array}$$

where *F* is a differential scale factor. As for our scheduling problem, the interim individuals *v*_i*_^g+1^ should be rounded to the nearest integer since decision variables *M*_*s*,*ij*_ and *M*_*h*,*j*_ must be positive integers, where *i* = 1,2,…, *n*, *j* = 1,2,…, *m*.

#### 4.2.3. Hybridization

The interim individuals *v*_i*_^g+1^ should be crossed with current individuals *x*_i*_^g^ in probability CR, where CR ∈ [0,1]. The proper individuals can be generated after hybridization. Set *U*_i*_^g+1^ = (*u*_i*1_^*g*+1^, *u*_i*2_^*g*+1^,…, *u*_i*D_^*g*+1^). *u*_i*1_^*g*+1^, *u*_i*2_^*g*+1^,…, *u*_i*D_^*g*+1^ is a feasible solution of decision variables. Consider

(18) $$\begin{array}{c} u_{i^{*}j^{*}}^{g+1}=\{\begin{array}{cc} v_{i^{*}j^{*}}^{g+1} & \text{ran}{\text{d}}_{i^{*}j^{*}}\leq \text{CR}\;\text{or}\;j=\text{rand}\left(i\right)\\ x_{i^{*}j^{*}}^{g} & \text{else} \end{array}\\ \;\;\;\;\;\left(i^{*}=1,2,\ldots ,\text{NP};j^{*}=1,2,\;,D\right), \end{array}$$

where CR is the cross rate. The larger the CR is, the more the *u*_i*j*_^g+1^ can be influenced by *v*_i*j*_^g+1^, which leads the algorithm to faster convergence with local optimization. In order to increase the performance of differential evolution algorithm, the auto-adapted cross rate was proposed. Let CR (*G*_*t*=0_) = CR_max⁡_. When the differential evolution algorithm in the fixed loop of evaluation does not improve significantly, CR can be automatically adapted according to

(19) $$\begin{array}{c} \text{CR}\left(G_{t+1}\right)=\{\begin{array}{cc} 0.95\text{CR}\left(G_{t}\right) & \text{if}\;0.95\text{CR}\left(G_{t}^{}\right)\geq \text{C}{\text{R}}_{\min}\\ \text{C}{\text{R}}_{\min} & \text{else,} \end{array} \end{array}$$

where CR_max⁡_ and CR_min⁡_ are the maximum and minimum crossover probabilities, respectively. *G* is the total evaluation number. *G*_*t*+1_ stands for evaluation value in cycle *t* + 1. The auto-adapted change of CR can improve performance of the whole algorithm and enhance the ability of global optimization algorithms.

#### 4.2.4. Selection

The fitness of candidate individual *U*_i*_^g+1^ should be evaluated after hybridization. The candidate individual *U*_i*_^g+1^ can be determined whether it replaces the current individuals *x*_i*_^g^ or not according to

(20) $$\begin{array}{c} x_{i^{*}}^{g+1}=\{\begin{array}{cc} U_{i^{*}}^{g+1} & \text{if}\;C_{\text{csc}}\left(T,U_{i^{*}}^{g+1}\right)\leq C_{\text{csc}}\left(T,x_{i^{*}}^{g}\right)\\ x_{i^{*}}^{g} & \text{else,} \end{array} \end{array}$$

where *C*_csc_(·) is the fitness function, which corresponds to the total cost of (11), and *T* = {*T*_1_, *T*_2_,…, *T*_*m*_}, where *T*_j_* (*j* = 1,2,…, *m*) can be calculated from (12). The process should be repeated and the best solution should be output corresponding to *x*_i*_^g+1^ and *T*.

## 5. Numerical Analysis

### 5.1. Parameters Setting

Numerical experiments are conducted to examine the computational effectiveness and efficiency of the proposed auto-adapted differential evaluation algorithm by comparing it with the method of decentralized decision. The parameters of the auto-adapted DE algorithm are as follows: *D* = *m* + *m*∗*n*, NP = *D*, *F*_min⁡_ = 0.2, *F*_max⁡_ = 0.6, CR_min⁡_ = 0.2, CR_max⁡_ = 0.8, and the maximum number of iterations* GenM* is set at 500 when *m* = 10 and *n* = 9,7, 5; * GenM* is set at 400 when *n* = 3 and *m* = 10,8; * GenM* is set at 300 when *n* = 3 and *m* = 6,4. The detailed settings for each test problem are as follows.

 
*A*_*s*,*i*_ is selected from uniform distribution *U* [200,300].
 
*h*_*s*,*i*_ is selected from uniform distribution *U* [1,3].
 
*F*_*s*,*ij*_ = 30 and *V*_*s*,*ij*_ is selected from uniform distribution *U* [10,20].
 
*A*_*h*_ = 50,  *h*_*h*,*i*_ = *α*_1_ · *h*_*s*,*i*_,  *α*_1_ = 0.8.
 
*F*_*h*,*j*_ = 5 and *V*_*h*,*j*_ is selected from uniform distribution *U* [1,6].
 If *w*_*ij*_ = 1, *d*_*ij*_ is selected from uniform distribution *U* [10,20]; otherwise, *d*_*ij*_ = 0.
 
*h*_*m*,*j*_ = *α*_2_ · (max⁡*h*_*s*,*i*_), *α*_2_ = 3.
 
*A*_*m*_ is selected from uniform distribution *U* [20,30].

### 5.2. Comparative Evaluations

**Figure 2:**
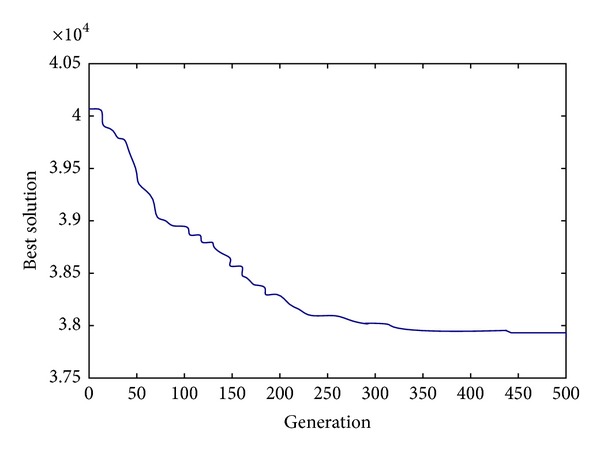
The evolution of best solution when *m* = 9, *n* = 10.

**Figure 3:**
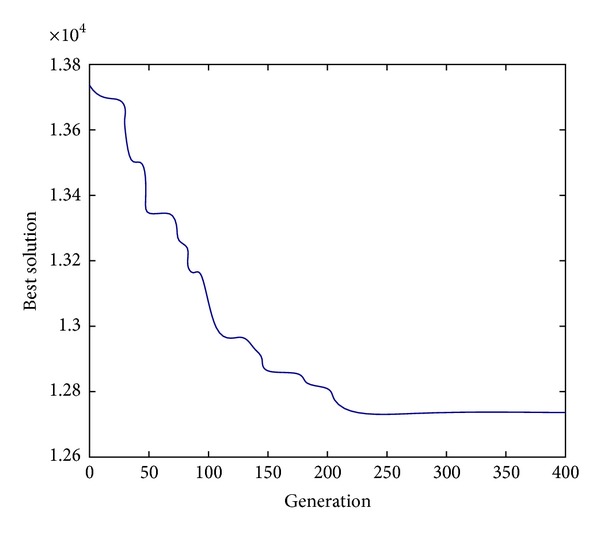
The evolution of best solution when *m* = 3, *n* = 10.

**Figure 4:**
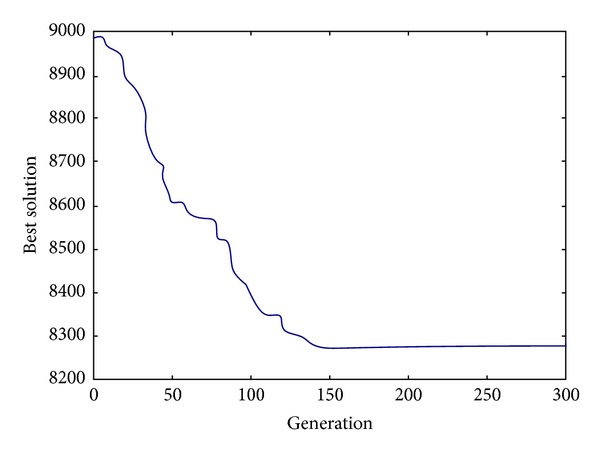
The evolution of best solution when *m* = 3, *n* = 6.

Figures 2, 3, and 4 show the evolution of best solution under 3 different cases, respectively, and we run the proposed auto-adapted DE algorithm under every case for 100 times and calculate its best solutions, worst solutions, means, and standard deviations; the result is shown in Table 1. The results of the auto-adapted DE algorithm and those of the method of decentralized decision are shown in Tables 2 and 3. In Table 2, we assume *n* = 10 and *m* = 9,7, 5,3. In Table 3, we assume *m* = 3 and *n* = 10,8, 6,4. In both the two tables, C_*s*,*i*_ denotes the cost of supplier *i*; C_*h*_ denotes the cost of Supply-Hub; C_*m*,*j*_ denotes the cost of manufacturer *j*; and C_sc_ denotes the cost of supply chain.  Table 4 shows the difference of every cost item in the context of joint decision and decentralized decision when *n* = 10.  Table 5 shows the difference of every cost item in context of joint decision and decentralized decision when *m* = 3.

From Figures 2, 3, and 4, it can be seen that the auto-adapted DE algorithm is convergent under these 3 cases; in fact, the algorithm is convergent under all these 7 cases in our numerical experiment; we only show the 3 figures due to the limited space. From Table 1 we can see that even under the case *m* = 9 and *n* = 10, the standard deviation is relatively small, so we can conclude that the auto-adapted DE algorithm is stable.

From Tables 2, 3, 4, and 5, we can obtain some conclusions as follows.

1. When suppliers, the Supply-Hub, and manufacturers make decisions as a whole, the total cost of supply chain can be reduced compared to the corresponding cost when they make decisions decentralized. Tables 4 and 5 reveal that the total cost of supply chain can be reduced by 5.4% at least, 7.4% at most.
2. When suppliers, the Supply-Hub, and manufacturers make decisions centralized, every supplier's cost decreases, but the Supply-Hub's cost and every manufacturer's cost increases, and the decreased cost is more than the increased one, so the total cost of supply chain can be reduced. We can see that the Supply-Hub's cost increases greatly in context of centralized decision-making from Tables 4 and 5, so the operator of the Supply-Hub may be not willing to make decisions centralized. In fact, suppliers always sell their products to the manufacturer on consignment under Supply-Hub mode. The inventory holding cost is paid by suppliers when their products are stored in the Supply-Hub, as every supplier's cost decreases greatly on the condition of centralized decision-making, so they are willing to pay the increased inventory holding cost.
3. The Supply-Hub's distribution interval and every supplier's distribution interval increase under centralized decision-making compared to the results obtained in the case of decentralized decision-making, but for supplier's order interval, some increase and others decrease. From Tables 2 and 3, it can be seen that every supplier's distribution interval and *M*_*h*,*j*_ increase in the context of centralized decision-making, so the Supply-Hub's distribution interval for every manufacturer also increases under this case.
4. From Table 2, it can be seen that in case of decentralized decision-making, all the suppliers' and Supply-Hub's decisions remain the same as the number of manufacturer increases, but under centralized decision-making, their decisions change as the number of manufacturer increases. This is because in the context of centralized decision-making, every decision maker considers the influence of his decision on others, and they optimize the whole supply chain collaboratively. Therefore, as the number of manufacturer increases, all the suppliers and the Supply-Hub change their optimal decisions.

## 6. Conclusions

This paper examines the collaborative scheduling model for the Supply-Hub consists of multiple suppliers and multiple manufacturers. We describe the basic operational process of the Supply-Hub and formulate the basic decision models. Given two different scenarios of decentralized system and collaborative system, we first consider the case that the Supply-Hub, the suppliers, and the manufacturers operate separately in their delivery quantities, production quantities, and order quantities. We next consider the collaborative mechanism, in which the Supply-Hub makes the entire decisions for all the suppliers and manufacturers. Furthermore, we offer the complexity analysis for the collaborative scheduling model and it turns to be proved NP-complete. Consequently, we propose an auto-adapted differential evolution algorithm. The numerical analysis illustrates that the performance of collaborative decision is superior to the decentralized decision. All these results demonstrate that the implementation of Supply-Hub can significantly reduce the operation cost in the assembly system, and thus improve the supply chain's overall performance.

## Figures and Tables

**Table 1:** Stability analysis of the auto-adapted DE algorithm.

*m*	*n*	Best value	Worst value	Mean	Standard deviation
9	10	38408	38633	38504	42
7	10	30160	30382	30304	38
5	10	21618	21783	21706	37
3	10	12786	12937	12855	31
3	8	10524	10666	10597	27
3	6	8267	8390	8311	26
3	4	5206	5418	5342	31

**Table 2:** The result of joint decision and decentralized decision when *n* = 10.

*m*	Joint decision							Decentralized decision						
	*M*_*h*,*j*_	*M*_*s*,*ij*_	*T*_*j*_	*C*_*s*,*i*_	*C*_*h*_	*C*_*m*,*j*_	*C*_sc_	*M*_*h*,*j*_	*M*_*s*,*ij*_	*T*_*j*_	*C*_*s*,*i*_	*C*_*h*_	*C*_*m*,*j*_	*C*_sc_
9	6 5 7 6 7 6 6 6 6	4 4 4 2 6 3 4 0 7 4 3 4 5 3 3 3 5 4 0 3 2 4 0 2 5 3 3 4 5 3 2 0 3 4 7 2 3 4 4 2 4 0 5 3 3 3 3 5 3 2 4 3 4 3 6 3 4 5 4 0 3 3 3 3 0 3 4 5 3 3 3 3 3 3 5 2 4 3 6 0 3 3 5 5 0 3 3 4 4 4	0.24 0.28 0.23 0.28 0.23 0.24 0.26 0.26 0.25	3021 2405 2921 3858 2803 2670 3273.5 3397 2800 2465	6177	243 254 266 264 221 226 256 241 206	37968	3 3 3 3 4 3 3 3 3	7 6 7 5 6 5 5 0 9 6 8 6 7 6 7 5 6 8 0 5 6 7 0 5 7 5 6 9 8 5 6 0 6 7 7 5 5 7 9 5 5 0 5 4 6 5 6 7 7 4 6 7 7 6 9 6 5 8 9 0 6 5 6 6 0 5 6 7 8 6 7 6 7 6 7 5 6 8 10 0 7 7 7 6 0 6 6 9 10 6	0.23 0.21 0.21 0.22 0.2 0.21 0.23 0.21 0.21	3334 2647 3151 4184 2939 3012 3545 3638 3032 2718	5801	243 245 266 258 219 224 253 234 203	40147

7	8 5 7 6 6 6 8	3 3 3 2 3 2 3 0 4 3 3 3 3 2 4 2 3 4 0 3 3 3 0 2 4 2 3 5 5 3 2 0 3 3 5 3 3 4 5 3 3 0 3 2 3 3 4 4 5 3 4 3 4 3 4 4 2 3 5 0 4 3 3 3 0 2 4 5 4 3	0.24 0.33 0.23 0.27 0.28 0.27 0.21	2392 1696.5 2139 2962 2333 2007 2464 2574 2193 2147	5157	243 269 267 262 230 230 255	29820	3 3 3 3 4 3 3	7 6 7 5 6 5 5 0 9 6 8 6 7 6 7 5 6 8 0 5 6 7 0 5 7 5 6 9 8 5 6 0 6 7 7 5 5 7 9 5 5 0 5 4 6 5 6 7 7 4 6 7 7 6 9 6 5 8 9 0 6 5 6 6 0 5 6 7 8 6	0.23 0.21 0.21 0.22 0.2 0.21 0.23	2638 1895 2355 3280 2517 2303 2719 2756 2365 2365	4779	243 245 266 258 219 224 253	31680

5	7 8 8 8 10	3 2 3 2 2 2 2 0 4 3 3 2 3 2 3 2 2 3 0 2 2 3 0 2 3 2 2 3 3 2 3 0 2 2 3 2 2 3 3 2 2 0 2 2 2 2 2 3 3 2	0.29 0.26 0.25 0.27 0.24	1600 995.5 1360.5 2170 1992 1429 1676 1556 1412 1754.5	3973	250 250 270 262 222	21172	3 3 3 3 4	7 6 7 5 6 5 5 0 9 6 8 6 7 6 7 5 6 8 0 5 6 7 0 5 7 5 6 9 8 5 6 0 6 7 7 5 5 7 9 5 5 0 5 4 6 5 6 7 7 4	0.23 0.21 0.21 0.22 0.2	1836 1151 1544 2457 2202 1704 1937 1721 1566 2017	3483	243 245 266 258 219	22848

3	8 7 9	3 2 3 2 3 2 2 0 5 2 3 2 3 2 3 2 2 3 0 2 2 3 0 2 2 2 2 5 4 2	0.26 0.29 0.22	955 996 617 1370 1294 968.5 1042 761 713 1016	2330	245 256 266	12786	3 3 3	7 6 7 5 6 5 5 0 9 6 8 6 7 6 7 5 6 8 0 5 6 7 0 5 7 5 6 9 8 5	0.23 0.21 0.21	1100.5 1151 702 1552 1377 1144 1204 834 776.5 1181	2039	243 245 266	13814

**Table 3:** The result of joint decision and decentralized decision when *m* = 3.

	Joint decision							Decentralized decision						
*n*	*M*_*h*,*j*_	*M*_*s*,*ij*_	*T*_*j*_	*C*_*s*,*i*_	*C*_*h*_	*C*_*m*,*j*_	*C*_sc_	*M*_*s*,*ij*_	*M*_*s*,*j*_	*T*_*j*_	*C*_*s*,*i*_	*C*_*h*_	*C*_*m*,*j*_	*C*_sc_
10	8 7 9	3 2 3 2 3 2 2 0 5 2 3 2 3 2 3 2 2 3 0 2 2 3 0 2 2 2 2 5 4 2	0.26 0.29 0.22	955 996 617 1370 1294 968.5 1042 761 713 1016	2330	245 256 266	12786	3 3 3	7 6 7 5 6 5 5 0 9 6 8 6 7 6 7 5 6 8 0 5 6 7 0 5 7 5 6 9 8 5	0.23 0.21 0.21	1100.5 1151 702 1552 1377 1144 1204 834 776.5 1181	2039	243 245 266	13814

8	8 8 8	2 2 2 2 2 2 2 0 3 2 2 2 3 2 2 2 2 2 0 2 2 2 2 3	0.3 0.28 0.28	939.5 979 600 1361 1234 963 1031 742	1980.5	223 234 236	10524	3 3 3	6 5 6 4 6 4 5 0 7 5 7 6 6 4 6 8 5 6 0 4 6 4 5 6	0.25 0.23 0.24	1085 1134.5 693 1536 1362 1127 1187 824	1703.5	220 229 234	11335

6	7 6 7	3 2 2 2 2 2 3 2 3 2 3 2 2 2 0 2 2 1	0.3 0.3 0.4	948 980 610 1365 1232 947	1570	204.5 205 205.5	8267	3 3 3	6 5 6 4 5 4 6 5 6 4 6 4 5 5 0 4 5 4	0.28 0.26 0.28	1066 1116 682 1512 1344 1105	1380	202 199 200	8806

4	6 6 8	2 2 2 2 2 2 2 2 1 2 0 1	0.4 0.4 0.5	892 966 594 1304	1057	162 155 154	5284	3 3 3	4 4 5 3 5 3 4 4 3 4 0 3	0.35 0.34 0.37	1030 1079 661.5 1470	895	159.5 152 150	5597

**Table 4:** The difference of every cost item when *n* = 10.

*m*	9	7	5	3
Δ*C*_s1_	−9.4%	−9.3%	−12.9%	−13.2%
Δ*C*_s2_	−9.1%	−10.4%	−13.5%	−13.5%
Δ*C*_s3_	−7.3%	−9.2%	−11.9%	−12.1%
Δ*C*_s4_	−7.8%	−9.7%	−11.7%	−11.7%
Δ*C*_s5_	−4.6%	−7.3%	−9.5%	−6%
Δ*C*_s6_	−11.4%	−12.9%	−16.1%	−15.3%
Δ*C*_s7_	−7.6%	−9.4%	−13.5%	−13.5%
Δ*C*_s8_	−6.6%	−6.6%	−9.6%	−8.8%
Δ*C*_s9_	−7.7%	−7.3%	−9.8%	−8.1%
Δ*C*_s10_	−9.3%	−9.2%	−13%	−14%
Δ*C*_*m*1_	0%	0%	2.9%	0.8%
Δ*C*_*m*2_	3.7%	9.8%	2%	4.5%
Δ*C*_*m*3_	0%	0.4%	1.5%	0%
Δ*C*_*m*4_	2.3%	1.6%	1.6%	
Δ*C*_*m*5_	0.9%	5%	1.4%	
Δ*C*_*m*6_	0.9%	2.7%		
Δ*C*_*m*7_	1.2%	0.8%		
Δ*C*_*m*8_	3%			
Δ*C*_*m*9_	1.5%			
Δ*C*_*h*_	6.5%	7.9%	14.1%	14.3%
Δ*C*_sc_	−5.4%	−5.9%	−7.3%	−7.4%

**Table 5:** The change of every cost item when *m* = 3.

*n*	10	8	6	4
Δ*C*_*m*1_	0.8%	1.4%	1.2%	1.3%
Δ*C*_*m*2_	4.5%	2.2%	3%	2%
Δ*C*_*m*3_	0%	0.9%	2.8%	2.7%
Δ*C*_s1_	−13.2%	−13.4%	−11.1%	−13.4%
Δ*C*_s2_	−13.5%	−13.7%	−12.2%	−10.5%
Δ*C*_s3_	−12.1%	−13.4%	−10.6%	−10.3%
Δ*C*_s4_	−11.7%	−11.4%	−9.7%	−11.3%
Δ*C*_s5_	−6%	−9.4%	−8.3%	
Δ*C*_s6_	−15.3%	−14.6%	−14.3%	
Δ*C*_s7_	−13.5%	−13.1%		
Δ*C*_s8_	−8.8%	−10%		
Δ*C*_s9_	−8.1%			
Δ*C*_s10_	−14%			
Δ*C*_*h*_	14.3%	16.3%	13.8%	18.1%
Δ*C*_sc_	−7.4%	−7.2%	−6.1%	−5.6%
